# Supplementary material for: Loss of STING in parkin mutant flies suppresses muscle defects and mitochondria damage
Source: PLoS Genet. 2023 Jul 13;19(7):e1010828. doi: 10.1371/journal.pgen.1010828 (PMC10368295; doi:10.1371/journal.pgen.1010828)
Supplement: S1 Fig — Related to Fig 1. (PDF) [file pgen.1010828.s001.pdf]

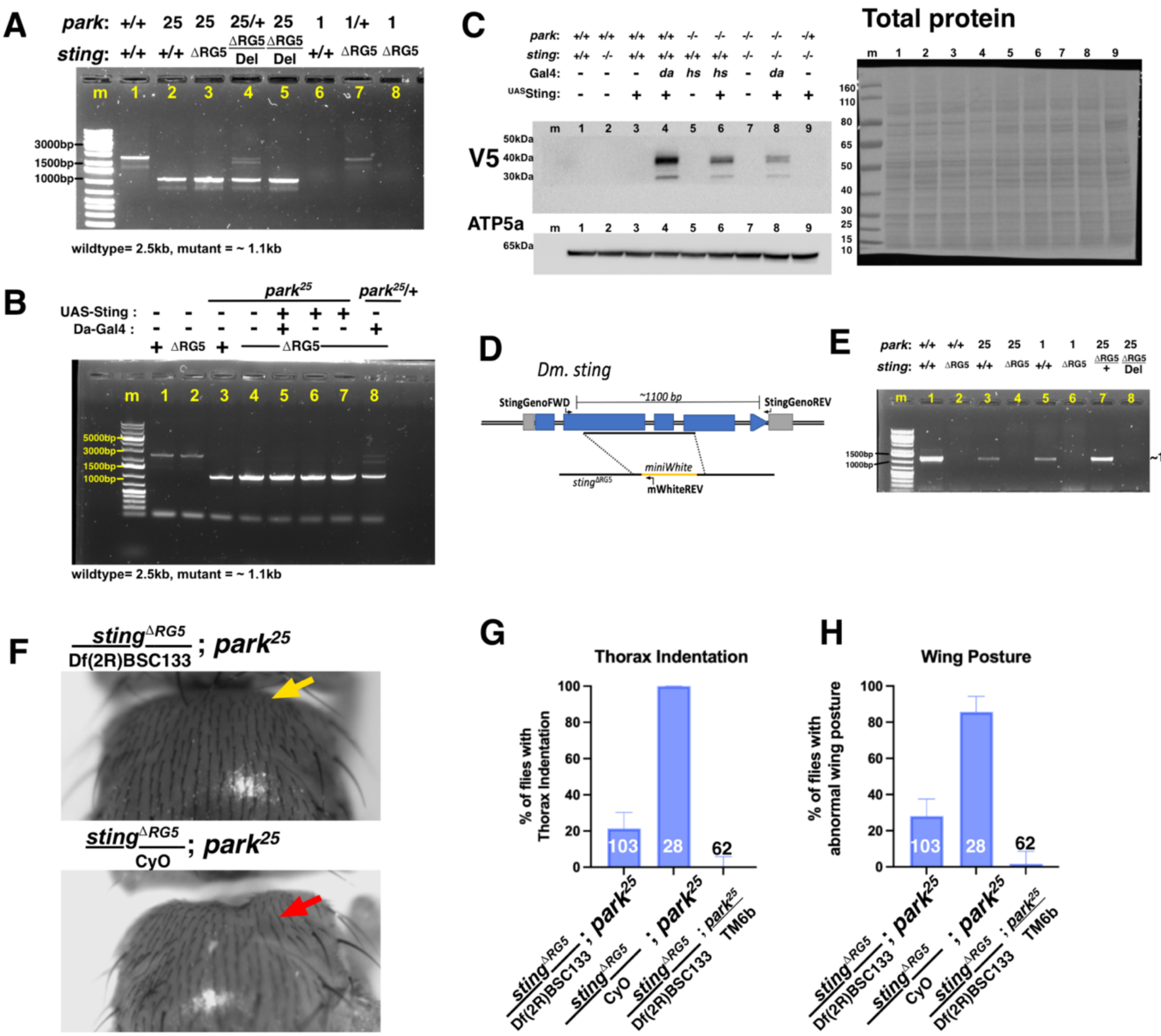

Fig S1- Verification of *parkin* and *sting* mutant alleles. (**A**) Example of DNA genotyping gel for verification of *parkin* allele status in key fly genotypes. Genomic DNA was prepared from 3-4 flies per genotype and subjected to PCR amplification with primers flanking the region deleted in the $park^{25}$ alleles. Note that this PCR will produce no product in homozygous $park^{1}$ mutant. See samples 6 and 8. (**B**) DNA genotyping gel for presence of the $park^{25}$ mutant allele in fly stocks containing recombined $park^{25}$ *DaGal4,* or $park^{25}$ UAS-STING-V5 alleles (**C**) Western blots for verification of V5-tagged STING in flies with combinations of either *Da*Gal4 (sample 4), *hs70*-Gal4 (samples 4 and 6), or the recombined $park^{25}$*,DaGal4* allele (samples 8) expressing UAS-Sting-V5. Relatively equal amounts of protein were loaded for each sample. (**D**) Schematic of the $sting^{\Delta RG5}$ allele and genotyping strategy. This PCR produces a band of 1145 base pairs in a wild-type allele. Due to the presence of the large *mwhite* replacement cassette in the ΔRG5 allele no PCR product is observed. (**E**) PCR Genotyping of single fly DNA samples for each of the indicated genotypes. Note that this amplification strategy cannot determine a heterozygous fly from a homozygous mutant. The eighth lane contains samples from the heteroallelic combination shown in panel C. (**F**). Flies resulting from crossing $sting^{\Delta RG5}$*;* $park^{25}$*/TM6b, hu* with flies containing a deficiency allele deleting the entire *sting* gene, *Df(2R)BSC133/CyO ;* $park^{25}$*/TM6b, hu.* The resulting *Df(2R)BSC133/CyO ;* $park^{25}$*/*$park^{25}$ or *Df(2R)BSC133/* $sting^{\Delta RG5}$ *;* $park^{25}$*/*$park^{25}$ progeny were examined for the severity of (**G**) thorax defects and (**H**) wing posture. The *Df(2R)BSC133/* $sting^{\Delta RG5}$ *;* $park^{25}$*/*$park^{25}$ showed similar suppression of the $park^{25}$ phenotypes as the homozygous $sting^{\Delta RG5}$*/*$sting^{\Delta RG5}$*;* $park^{25}$*/*$park^{25}$ animals.
