# Supplementary material for: Loss of STING in parkin mutant flies suppresses muscle defects and mitochondria damage
Source: PLoS Genet. 2023 Jul 13;19(7):e1010828. doi: 10.1371/journal.pgen.1010828 (PMC10368295; doi:10.1371/journal.pgen.1010828)
Supplement: S2 Fig — Related to Fig 1. (PDF) [file pgen.1010828.s002.pdf]

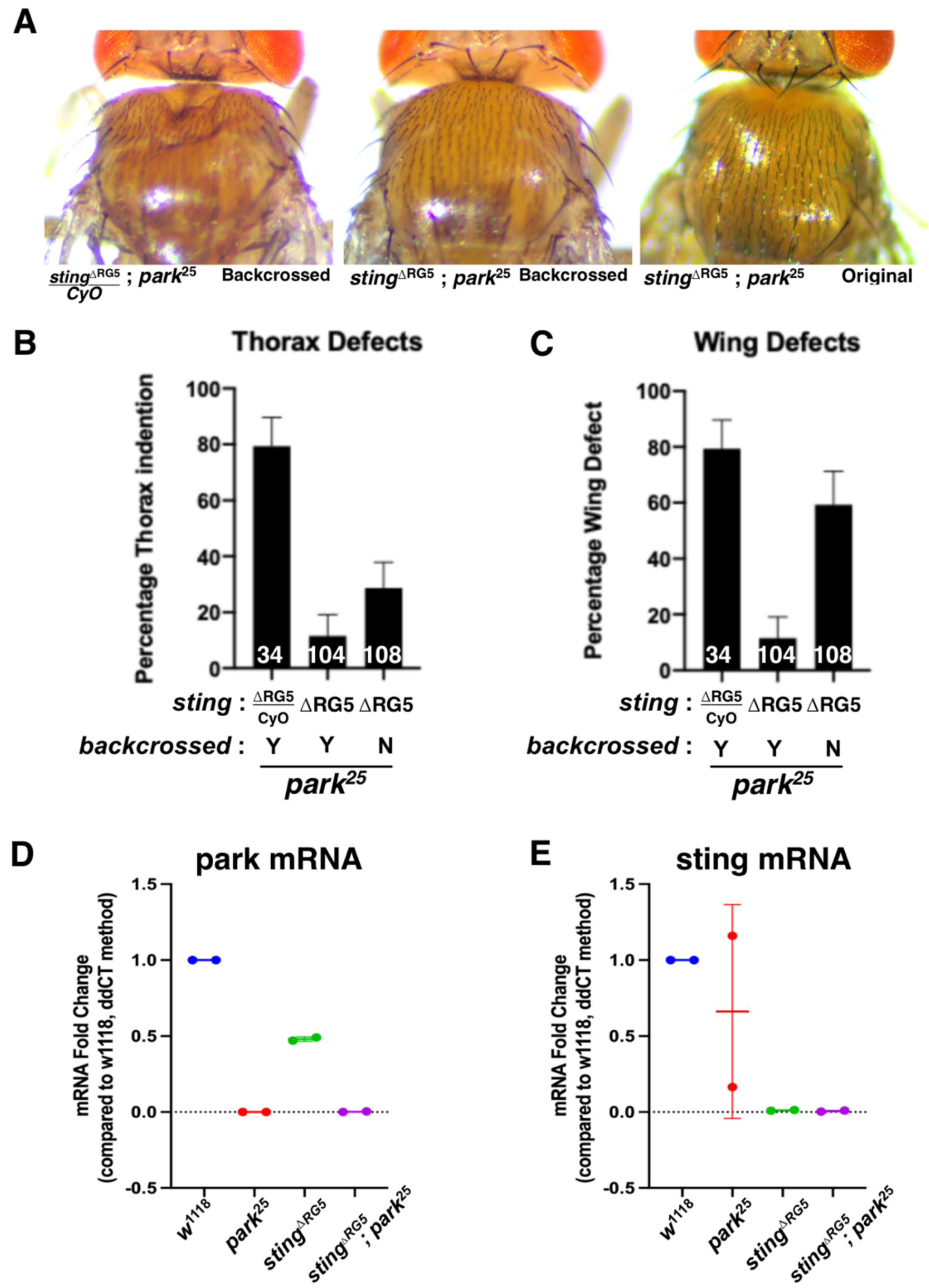

Fig S2- Analysis and validation of an independent $sting^{\Delta RG5}$;$park^{25}$ stock. (**A**) Example images of thoraces of $sting^{\Delta RG5}$; $park^{25}$ and heterozygous $sting^{\Delta RG5}$/CyO; $park^{25}$ siblings after backcrossing to a w^{1118} stock. Also shown is an example of a $sting^{\Delta RG5}$; $park^{25}$ fly from the stock gifted from the Whitworth lab, after being raised for 2 generations in our food and incubator. Note that the thorax indentation defect is mostly suppressed, and the wing posture defects show mild suppression. Quantification of the thorax indication and wing posture defects are shown in (**B**) and (**C**), respectively. In both graphs, bars represent the percentage of flies displaying the indicated phenotype, numbers indicate the number of flies scored per genotype, and the error bars represent the 95% confidence interval for the population proportion. (**D and E**). Relative amounts of mRNA following RT-qPCR for both (**D**) *parkin* and (**E**) *sting*, from two independent cDNA samples of control flies, and $sting^{\Delta RG5}$; $park^{25}$ after backcrossing to w^{1118}.
