# Supplementary material for: Loss of STING in parkin mutant flies suppresses muscle defects and mitochondria damage
Source: PLoS Genet. 2023 Jul 13;19(7):e1010828. doi: 10.1371/journal.pgen.1010828 (PMC10368295; doi:10.1371/journal.pgen.1010828)
Supplement: S3 Fig — Related to Fig 4. (PDF) [file pgen.1010828.s003.pdf]

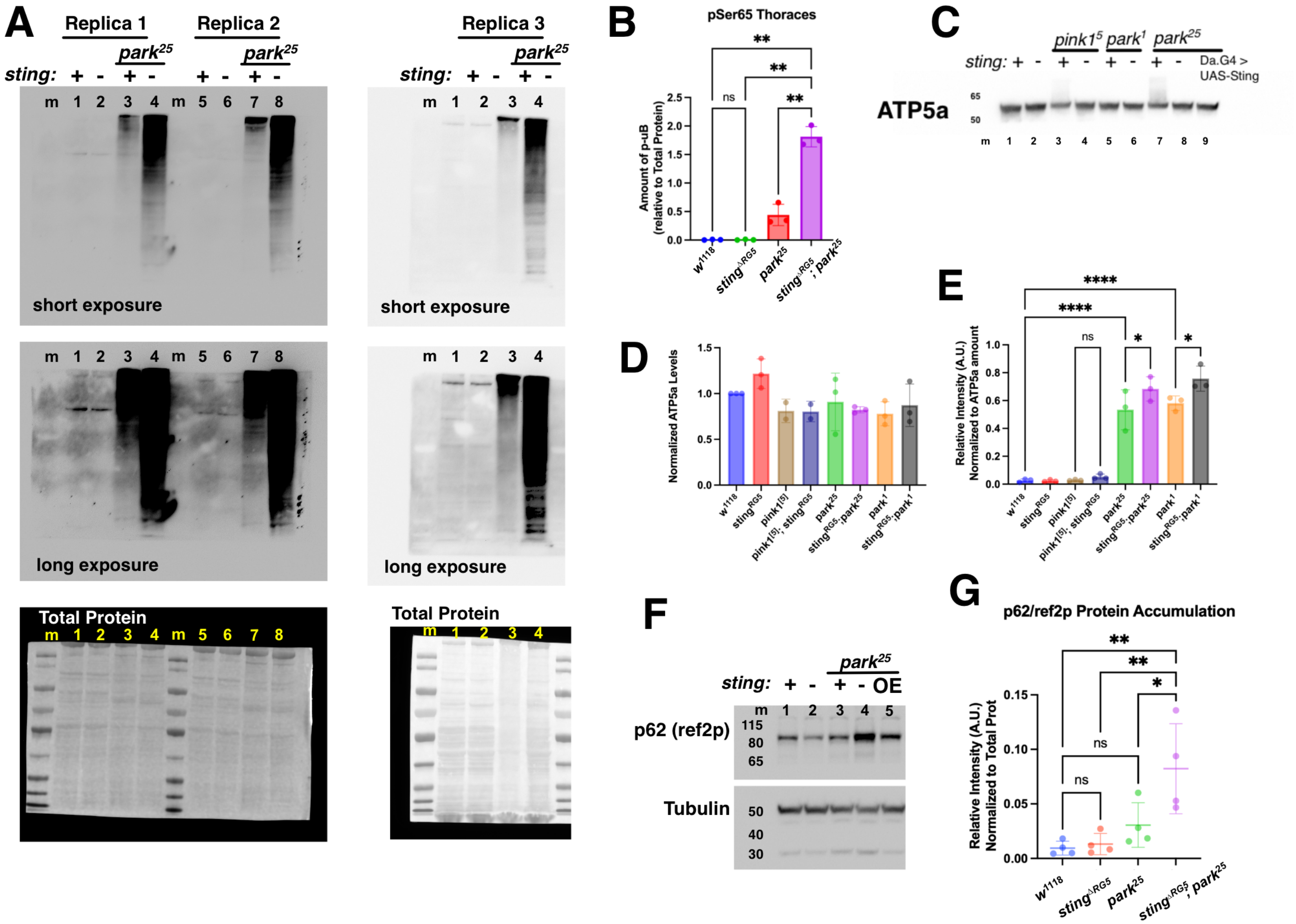

**Fig S3- Analysis of Sting-regulated innate immunity pathways and phosphorylated Ubiquitin measurements from mutant thorax samples.** (**A**) Western blots for pSer65-Ub using protein samples derived from dissected thoraces of the indicated genotypes. Three independent biological replicas were generated and tested. (**B**) Quantification of blots from pSer65-Ub western blots. Statistical significance was determined with a 1-way ANOVA test, followed by Dunnet's multiple comparison's testing. ** indications a p-value less than 0.01. Bars represent mean and standard deviation. (**C**) Example Western blot for ATP5a on total protein samples isolated from 5 whole flies. See methods for antibody and protocol details. (**D**) Quantification of western blot band intensities for ATP5a in 3 biological replica experiments (N= 2 for *pink1[5]* and *pink1[5]; sting[RG5]* samples). ImageJ was used to assess band intensities, then bands were normalized to total protein levels on the same blot. For comparing between samples, each biological sample was normalized to a corresponding wild-type control sample. Bars represent mean and standard deviation. (**E**) Normalization of the phosphor-Ub data displayed in Fig 4B to the relative mean amount of ATP5a levels in each of the indicated genotypes, to approximate the relative amount of pUb to total mitochondria. Statistical significance was determined with a 1-way ANOVA test, followed by Bonferroni's multiple comparison's testing. * indications a p-value less than 0.05. Bars represent mean and standard deviation. (**F**) Western blotting against whole fly samples for the autophagy cargo adaptor p62 (ref2p). (**G**) Quantification of p62 blots as presented in F. N= 3 for each genotype, and for each biological replica, the band intensities were normalized to each lane's total protein stain. Bars represent mean and standard deviation. For statistical significance, a 1-way ANOVA test, followed by Bonferroni's multiple comparison's tests were performed. ** = p-value less than 0.01 and * = p-value less than 0.05.
