# Supplementary material for: Loss of STING in parkin mutant flies suppresses muscle defects and mitochondria damage
Source: PLoS Genet. 2023 Jul 13;19(7):e1010828. doi: 10.1371/journal.pgen.1010828 (PMC10368295; doi:10.1371/journal.pgen.1010828)
Supplement: S4 Fig — Related to Fig 5. (PDF) [file pgen.1010828.s004.pdf]

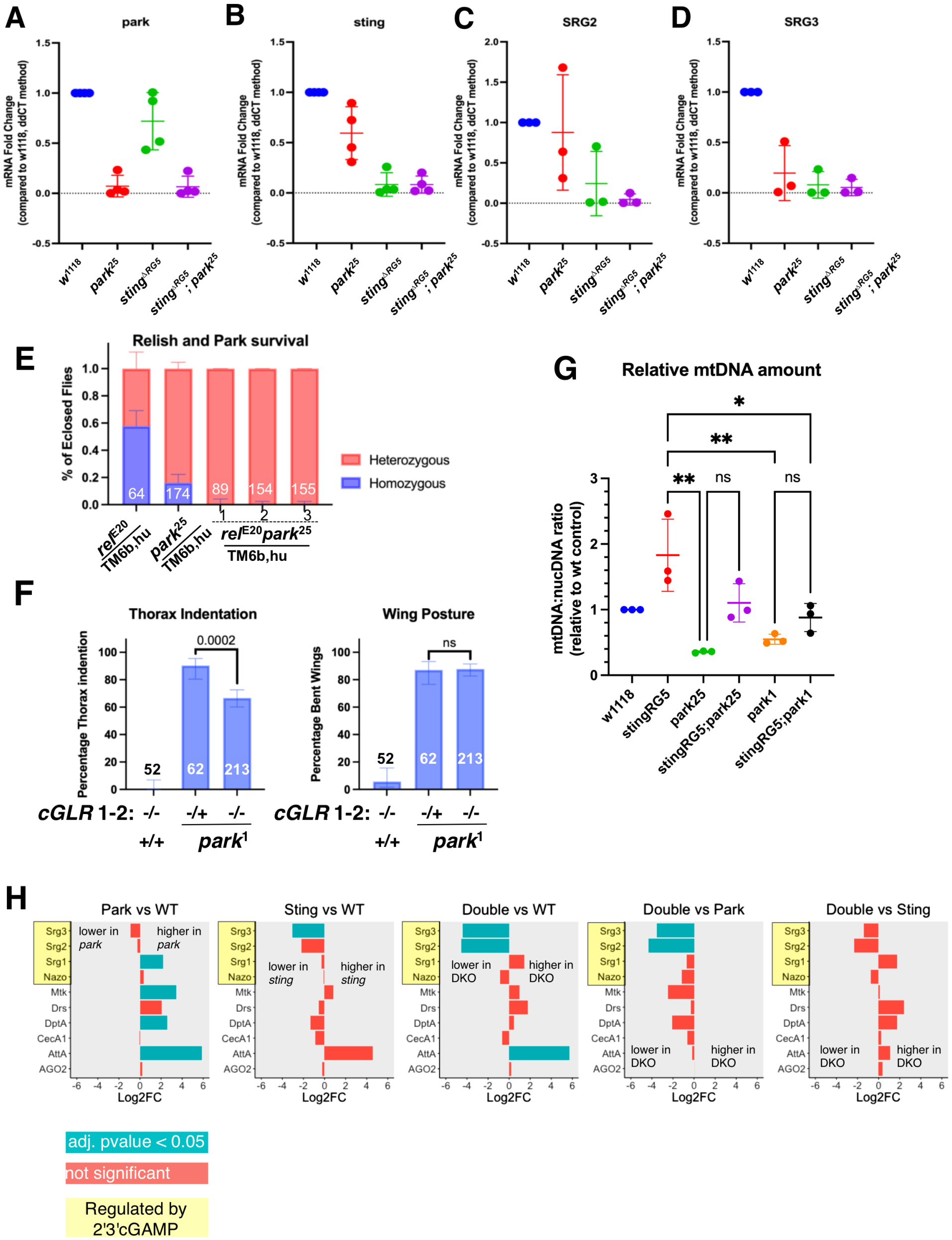

Fig S4- Analysis of Sting-regulated innate immunity in *park* mutants Relative amounts of mRNA following RT-qPCR for the indicated gene, from at least 3 independent cDNA samples, each consisting of 10 flies of the specified genotypes. (**A**) *park* (**B**) *sting* (**C**) *SRG2* (CG42825) (**D**) *SRG3* (CG33926). Bars represent mean and standard deviation. Each point represents normalized results of a biological replica. All expression was normalized first to an internal housekeeping gene, then to the wild-type control. **(E)** Quantification of synthetic lethality in a *relish*^{E20} and *park*^{25} mutant background. No homozygous flies are observed in 3 lines originating from single males carrying a recombined *rel*^{E20} *park*^{25} allele. (**F**) Mutations in two STING activators, cGLR1 and cGLR2, do not severely suppress *park*^{1} mutant phenotypes. There is a slight reduction in the penetrance of the thorax indentation, however no effect was seen on the wing posture severity. (**G**) mtDNA copy number in *parkin* mutant animals is lower than wild-type and *sting* mutant flies show elevated mtDNA copies. mtDNA levels were assayed by multiplex qPCR on three independent biological total DNA samples (text continues next page).
Each sample was normalized to a nuDNA reference control. Data shown is normalized copy number compared to a wild-type control in each biological replica. (**H**) Normalized expression levels of genes previously studied as STING-regulated target genes. The anti-viral genes highlighted in yellow have been previously reported to respond to 2’3cGAMP in a STING-dependent manner. The non-highlighted genes act downstream of the STING/IMD/Relish pathway but are also regulated by the PGRP-LC/IMD/Relish and the Toll/MyD88 pathways. For an anti-viral gene control, expression levels of Argonaute2 (AGO2) are included. Data is shown as Log2 Fold Change. Significance was determined with an adjusted FDR cutoff of 0.05.
