# Supplementary material for: Loss of STING in parkin mutant flies suppresses muscle defects and mitochondria damage
Source: PLoS Genet. 2023 Jul 13;19(7):e1010828. doi: 10.1371/journal.pgen.1010828 (PMC10368295; doi:10.1371/journal.pgen.1010828)
Supplement: S5 Fig — Related to Fig 5. (PDF) [file pgen.1010828.s005.pdf]

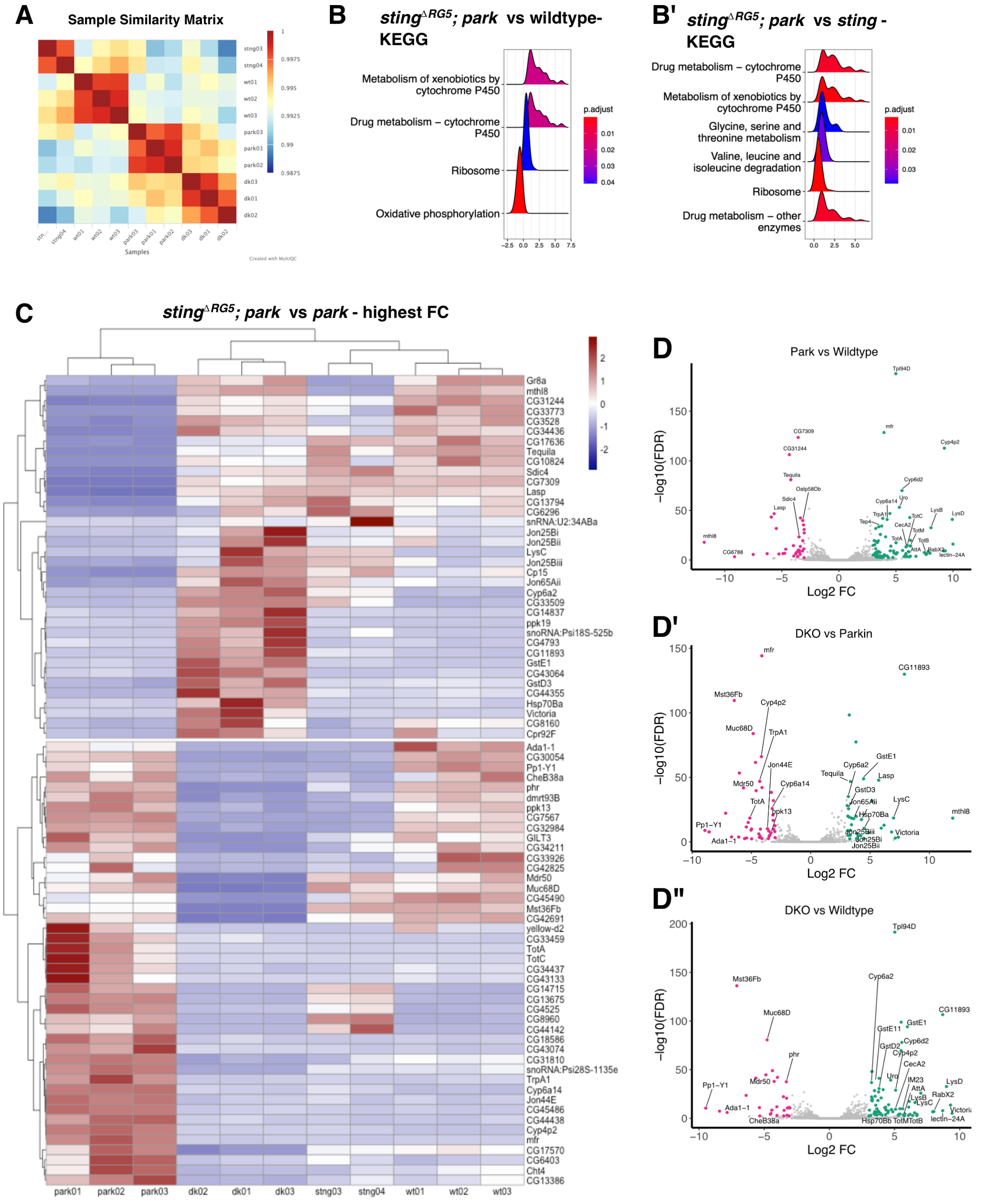

Fig S5 – RNA Seq experiment details and additional sample comparisons. (**A**) Sample similarity matrix, generated by MultiQC, to display sample validity and homogeneity. (**B**) Ridgeplot graphs indicate the distribution of expression levels for significantly enriched KEGG sets for analysis of (**B**) *sting*$^{\Delta RG5}$*; park*^{25} to wild-type samples, and (**B'**) *sting*$^{\Delta RG5}$*; park*^{25} to *sting*$^{\Delta RG5}$ mutants. Results are from GSEA with the KEGG classifications network, with an adjusted p-value (BH) cutoff of 0.05. (**C**) Heatmap of normalized expression counts, normalized by Z-score, of the top differentially expressed genes between the *sting*$^{\Delta RG5}$*; park*^{25} and *park*^{25} samples. (**D**) Volcano plots of the log2 fold-change and log10 adjusted FDR for all transcripts in the indicated comparison: **D-** *park*^{25} and wild-type, **D'-** *sting*$^{\Delta RG5}$*; park*^{25} and *park*^{25}, **D"-** *sting*$^{\Delta RG5}$*; park*^{25} and wild-type. Some of the highest enriched genes are labeled. Green indications significantly enriched transcripts and magenta labels suppressed transcripts, both at cut-off of log2FC greater than 3 and adjusted FDR less than 0.005.
