# Supplementary material for: Loss of STING in parkin mutant flies suppresses muscle defects and mitochondria damage
Source: PLoS Genet. 2023 Jul 13;19(7):e1010828. doi: 10.1371/journal.pgen.1010828 (PMC10368295; doi:10.1371/journal.pgen.1010828)
Supplement: S1 Table — (DOCX) [file pgen.1010828.s006.docx]

**Table S1: Fly genotypes for all Figures**

| **Location** | **Genotype** |
| --- | --- |
| **Fig 1** | |
| A,B, & C | *w^1118^* |
|  | *w^1118^; sting^∆RG5^* |
|  | *w^1118^;; park^25^* |
|  | *w^1118^; sting^∆RG5^; park^25^* |
|  | *w^1118^; ; park^1^* |
|  | *w^1118^; sting^∆RG5^; park^1^* |
| D | *w^1118^* |
|  | *w^1118^; sting^∆RG5^* |
|  | *w^1118^;; park^25^* |
|  | *w^1118^; sting^∆RG5^; park^25^* |
|  | *w^1118^;; park^1^* |
|  | *w^1118^; sting^∆RG5^; park^1^* |
| E, F, & G | *w*, pink1^[5]^* |
|  | *w*, pink1^[5]^; sting^∆RG5^* |
|  | *w*, pink1^[B9]^* |
|  | *w*, pink1^[B9]^; sting^∆RG5^* |
| **Fig 2** | |
| A | *w^1118^* |
| B | *w^1118^; sting^∆RG5^* |
| C | *w^1118^;; park^25^* |
| D | *w^1118^; sting^∆RG5^; park^25^* |
| E | *w^1118^;; park^1^* |
| F | *w^1118^; sting^∆RG5^; park^1^* |
| **Fig 3** | |
| A | *w^1118^; sting^∆RG5^; park^25^ DaGal4 /park^25^ UAS-Sting* |
|  | *w^1118^; sting^∆RG5^; park^25^ UAS-Sting/park^25^* |
| B,C & D | *w^1118^; ; UAS-Sting/ Da.Gal4* |
| B,C & E | *w^1118^; hs70Gal4 ; park^25^* |
| B,C & F | *w^1118^; hs70Gal4; park^25^ UAS-Sting/park^25^* |
| B,C | *w^1118^; sting^∆RG5^; park^25^ UAS-Sting/TM6B* |
| B,C & G | *w^1118^; sting^∆RG5^; park^25^ UAS-Sting/park^25^* |
| B,C & H | *w^1118^; sting^∆RG5^; park^25^ DaGal4 /park^25^ UAS-Sting* |
| **Fig 4** | |
| A & B | *w^1118^* |
|  | *w^1118^; sting^∆RG5^* |
|  | *w^1118^;; park^25^* |
|  | *w^1118^; sting^∆RG5^; park^25^* |
| C & D | *w^1118^* |
|  | *w^1118^; sting^∆RG5^* |
|  | *w*, pink1^[5]^* |
|  | *w*, pink1^[5]^; sting^∆RG5^* |
|  | *w^1118^;; park^25^* |
|  | *w^1118^; sting^∆RG5^; park^25^* |
|  | *w^1118^; ; park^1^* |
|  | *w^1118^; sting^∆RG5^;park^1^* |
| **Fig 5** | |
| E | *w^1118^* |
|  | *w^1118^; sting^∆RG5^* |
|  | *w^1118^;; park^25^* |
|  | *w^1118^; sting^∆RG5^; park^25^* |
| **Fig 1 (S1)** | |
| A | *w^1118^* |
|  | *w^1118^; ; park^25^* |
|  | *w^1118^; sting^∆RG5^; park^25^* |
|  | *w^1118^; sting^∆RG5^/stingDEF ; park^25^/TM6B* |
|  | *w^1118^; sting^∆RG5^/stingDEF ; park^25^* |
|  | *CyO/Kr^lf1^; park^1^* |
|  | *w^1118^; sting^∆RG5^; park^1^/TM6B* |
|  | *w^1118^; sting^∆RG5^; park^1^* |
| B | *w^1118^* |
|  | *w^1118^; sting^∆RG5^* |
|  | *w^1118^; ; park^25^* |
|  | *w^1118^; sting^∆RG5^; park^25^* |
|  | *w^1118^; sting^∆RG5^; park^25^ DaGal4 /park^25^ UAS-Sting* |
|  | *w^1118^; sting^∆RG5^; park^25^ DaGal4 /park^25^ UAS-Sting* |
|  | *w^1118^; sting^∆RG5^; park^25^ UAS-Sting/park^25^* |
|  | *w^1118^; sting^∆RG5^; park^25^ DaGal4 /TM6B* |
| C | *w^1118^* |
|  | *w^1118^; sting^∆RG5^* |
|  | *w^1118^; ; UAS-Sting/ TM6B* |
|  | *w^1118^; ; UAS-Sting/ Da.Gal4* |
|  | *w^1118^; hs70Gal4 ; park^25^* |
|  | *w^1118^; hs70Gal4; park^25^ UAS-Sting/park^25^* |
|  | *w^1118^; sting^∆RG5^; park^25^ UAS-Sting/park^25^* |
|  | *w^1118^; sting^∆RG5^; park^25^ DaGal4 /park^25^ UAS-Sting* |
|  | *w^1118^; sting^∆RG5^; park^25^ UAS-Sting/TM6B* |
| E | *w^1118^* |
|  | *w^1118^; sting^∆RG5^* |
|  | *w^1118^; ; park^25^* |
|  | *w^1118^; sting^∆RG5^; park^25^* |
|  | *w^1118^; CyO/Kr^lf1^; park^1^* |
|  | *w^1118^; sting^∆RG5^;park^1^* |
|  | *w^1118^; sting^∆RG5^/CyO; park^25^* |
|  | *w^1118^; sting^∆RG5^/stingDEF; park^25^* |
| F,G,H | *w^1118^; sting^∆RG5^/stingDEF; park^25^* |
|  | *w^1118^; sting^∆RG5^/CyO; park^25^ (Sibling from cross)* |
|  | *w^1118^; sting^∆RG5^/stingDEF; park^25^/TM6B (Sibling from cross)* |
| **Fig 2 (S2)** | |
| A, B, C | *w^1118^; sting^∆RG5^CyO; park^25^* |
|  | *w^1118^; sting^∆RG5^; park^25^* |
|  | *w*; sting^∆RG5^; park^25^* |
| D&E | *w^1118^* |
|  | *w^1118^; ; park^25^* |
|  | *w^1118^; sting^∆RG5^* |
|  | *w^1118^; sting^∆RG5^; park^25^* |
| **Fig 3 (S3)** | |
| A & B | *w^1118^* |
|  | *w^1118^; ; park^25^* |
|  | *w^1118^; sting^∆RG5^* |
|  | *w^1118^; sting^∆RG5^; park^25^* |
| C, D, and E | *w^1118^* |
|  | *w^1118^; sting^∆RG5^* |
|  | *w*, pink1^[5]^* |
|  | *w*, pink1^[5]^; sting^∆RG5^* |
|  | *w^1118^;; park^1^* |
|  | *w^1118^; sting^∆RG5^; park^1^* |
|  | *w^1118^; ; park^25^* |
|  | *w^1118^; sting^∆RG5^;park^25^* |
|  | *w^1118^; sting^∆RG5^; park^25^ DaGal4 /park^25^ UAS-Sting* |
| F & G | *w^1118^* |
|  | *w^1118^; sting^∆RG5^* |
|  | *w^1118^;; park^25^* |
|  | *w^1118^; sting^∆RG5^; park^25^* |
|  | *w^1118^; sting^∆RG5^; park^25^ DaGal4 /park^25^ UAS-Sting* |
| **Fig 4 (S4)** | |
| A,B,C,D | *w^1118^* |
|  | *w^1118^; ; park^25^* |
|  | *w^1118^; sting^∆RG5^* |
|  | *w^1118^; sting^∆RG5^; park^25^* |
| E | *w^1118^ ; ; rel^E20^/TM6B -*or- *rel^E20/E20^* |
|  | *w^1118^ ; ; park^25^/TM6B -*or- *park^25/25^* |
|  | *w* ; ; relish^E20^ park^25^/ TM6B- line 1* |
|  | *w* ; ; relish^E20^ park^25^/ TM6B - line 2* |
