# Supplementary material for: Loss of STING in parkin mutant flies suppresses muscle defects and mitochondria damage
Source: PLoS Genet. 2023 Jul 13;19(7):e1010828. doi: 10.1371/journal.pgen.1010828 (PMC10368295; doi:10.1371/journal.pgen.1010828)
Supplement: Attachment — Submitted filename: Moehlman et al. Response to reviewers.docx [file pgen.1010828.s010.docx]

***Response to Reviewers***

**Comments to the Authors:**
**Please note here if the review is uploaded as an attachment.**

Reviewer #1: I was excited when I first read this manuscript by Dr. Moehlman. In a way, it validates some unpublished phenomena we saw in pilot experiments (mentioned later). This manuscript attempts to address the interesting and critical question of whether cGAS/STING-mediated innate immune pathways affect the progression of PINK1-Parkin-associated Parkinson's disease. In mouse PD models, mitochondrial damage leads to the release of DAMPs that activate the cGAS/STING pathway. However, in the previous article published by Whitworth lab on the relationship between STING pathway and PINK1/Parkin (Lee et al., 2020, Sci Rep), they did not observe obvious genetic interaction. To be honest, we felt a little disappointed and confused. Because in our pilot experiments, we found in some pilot experiments that activating PP2A can maintain those dopaminergic neurons that would be degenerated in parkin mutants. And, in a recent paper by us and our collaborators (Ho et al., 2023, JCI), we found that PP2A inactivation can stimulate the activity of the cGAS/STING pathway and vice versa. It means, PP2A overexpression inhibits STING activity then rescues parkin. Given that both the Whitworth lab and the Youle lab have long-established reputations in mitophagy research, I think differences in genetic background are probably responsible for the observed differences in phenotypes.

Overall, I support the publication of this paper: on the one hand, this kind of academic debate is beneficial to the mitophagy society; on the other hand, this is a well-designed study, especially the genetics part (Fig. 1&2) . But I have a few questions and suggestions that I hope the author can consider as appropriate in their revision:

We appreciate the positive and constructive comments from the reviewer. In our revised manuscript, we have addressed the reviewer comments with new experiments and text changes as described below in our point by point reply. We have also further expanded on the role of *pink1* in these animals.

1) Does overexpression of STING in parkin mutants enhance the mutant phenotype and mitochondrial damage?

We assessed this possibility by generating flies with UAS-Sting overexpression in both a wild-type parkin (*Da*.Gal4/UAS-*sting*-V5) and a *park25* mutant background (*hs70.*Gal4; *park*^25^/UAS-*sting*, *park*^25^). Expression of Sting in these backgrounds was verified with western blotting (see Figure S1 Panel C). Expression of Sting in a wild-type background yielded no phenotypes. Overexpression of Sting in the *park*^25^ flies minimally increased the penetrance of the thorax and wing phenotypes (See revised figure 3, panels B and C), but did not affect the mitochondria morphology in the *hs70.*Gal4; *park*^25^/UAS-*sting*, *park*^25^ flies compared to *park^25^* mutants (See Figure 3 Panels E & F).

2) In addition to the signals already detected, have the authors thought about directly detecting DAMPs produced by mitochondria by using ddPCR? I think this should be a relatively direct evidence.

To assess this, we utilized qPCR on purified DNA samples from these flies. We used bulk collection of total DNA, normalized the amount of DNA template added to each qPCR reaction, and probed for mtDNA and nuclear DNA using a TaqMan-based probe assay. Our results indicate that in both *parkin* mutants there is a severe reduction in the relative amount of mitochondrial DNA compared to the wild-type controls. Our experiments suggest that mtDNA copy number is slightly elevated in *sting* mutants, and that deletion of *sting* in the *park* null flies (either allele) restores the relative mtDNA copy number to similar to wild-type (new Figure S4G). These results are similar to what we have observed for mitochondrial morphology and for apoptosis in these double mutant animals. These results support the hypothesis that *parkin* mutants experience mitochondria damage and cell death, but these phenomena are not due to elevated mtDNA levels in flies.

3) I think the determination of the GST pathway in Fig. 4 is a bit far-fetched. Of course, Fig.4A, 4B provide good support. But I feel that the 2-fold increase in GST activity shown in Fig. 4E does not necessarily provide a sufficient rationale for the previously observed rescues. In addition, if overexpressing GST or activating the GST pathway in parkin mutants, can defects be rescued as well as in STING KO? Or, if GST is knocked down in STING; park double mutant flies, will the previous rescue reverse? I think these genetic experiments can help to confirm whether GST is one of the most important downstream pathways activated.

We appreciate the feedback about the GST assays from the reviewer. Indeed, overexpression of GST genes has already been published to improve *parkin* or *pink1* mutant phenotypes see references and new text on lines 297-301. The suggested loss-of-function genetic experiments involving three (or more) mutant alleles would be ideal for testing this hypothesis, but due to the multiple genes involved and the possibility for many unknown interactions, we feel that these complex experiments are beyond the scope of this project. We revised the discussion and text to reflect that this is one of several possible hypotheses and remains unproven (See lines 308 - 311) Regarding the GST activity assay, these were performed on young adult flies post-eclosion, where the developmental phenotypes in the IFM were already present or absent. The contribution of the GST enzymes to suppressing the phenotypes may occur during specific developmental timepoints, which we did not assay. We toned down some of the discussions of GST in this context (lines 300-311).

4) I think the discussion of PINK1 in the article is not particularly sufficient, and I hope it can be strengthened.

We have expanded our discussion of Pink1 and included novel data from a second cross with a separate *pink1* mutant allele. See our response to Reviewer number 2 for additional details and the newly expanded Figure 1. The conclusion from this data is that loss of *sting* partially affects the phenotype of *pink1[b9]* mutants, but not to the extent that it suppresses loss of *parkin*. This suggests a similar role in mitigating mitochondria damage, but that there also remains *pink1-*dependent but *parkin-*independent factors in the pathology of the mitochondrial phenotypes.

I think this is a good manuscript and should be weighed positively.

Reviewer #2: This manuscript reports a genetic interaction between the Parkinson’s disease (PD) gene parkin and STING, which encodes a key component of an important evolutionarily conserved pathway of innate immunity. Parkin mediates selective autophagy of stressed mitochondria (mitophagy) and an important question in the field of PD relates to the consequences of the accumulation of damaged mitochondria in the absence of Parkin. One hypothesis that received experimental support is that stressed mitochondria release molecules that are sensed by the innate immune system and trigger inflammation. Indeed, this group previously reported that inhibition of STING in a mouse model deficient for parkin prevented inflammation, motor defect and neurodegeneration. In mammals, STING is activated by a cyclic dinucleotide second messenger produced by the enzyme cGAS, which acts as a receptor for cytosolic DNA, including released mitochondrial DNA. Here, the authors address the relevance of the interaction between parkin and STING in the drosophila model and report that, as in mice, loss of STING suppresses the muscle defects and mitochondria damage observed in parkin mutant flies. The results presented are convincing and novel. Of note, they contradict a previous study that concluded to a lack of interaction between parkin and STING. It is however not clear to me if this information is of sufficient novelty to justify publication in PLoS Genetics, especially given that some of the results are surprising (lack of interaction between pink1 and STING) and that the assets of the drosophila model are not really exploited to bring the field further in the understanding of the interaction between inflammation and PD. Addressing the comments listed below may make this manuscript a better candidate for publication in PLoS Genetics.

1) Lane 110: I note that a single allele of pink1 is used. Are other alleles available? My concern here is the issue of the genetic background masking the interaction with STING in one of the park[25] mutant lines. Has this pink1 line been isogenized in the same genetic background as the parkin mutants?

We appreciate the concerns about *pink1* and agree that it is interesting why a rescue similar to that of the *parkin* double mutants was not observed. The genetic background of the *pink1[5]* flies was established by outcrossing to the same *w*[1118] stock used for backcrossing the *parkin* stocks. Note that after the first cross*,* freely recombining *pink1[5]/+* females were backcrossed to a *FM6/y;* CyO/+ male to ensure the X-chromosome *pink1[5]* allele was recovered. From there, multiple *pink1[5]/FM6; +/CyO* flies were crossed with the *sting[RG5]* allele to generate the *pink1[B9]/FM6; sting[RG5]/CyO* candidate lines then PCR and phenotyping was used to double check *pink1* status.

We have since obtained the *pink1[B9]* allele and outcrossed it into the *FM6/y; stingRG5/CyO* background for three generations to obtain *pink1[B9]/y; sting[RG5]* males, so this will be approximately the same background as the *pink1[B9]/y; sting[RG5]* animals, except for the X-chromosome. X-chromosome recombination and verification of mutants would be challenging in the timeframe of this revision, so instead we maintained it with the FM6 balancer. In these animals, we found a small but significant suppression in the penetrance of the thorax indention phenotypes (See revised Figure 1, Panels E and F) in both *pink1[5] and pink1[B9] mutants*, but the wing posture defects were unaffected. Additionally, in our hands, neither of the *pink1* mutants displayed a climbing defect at five days post-eclosion, so we did not assess climbing in the *pink1; stingRG5* animals in the same manner as was done for *parkin* flies. We have revised the discussion of *pink1* to not rule out a role for *sting,* but our data and other published work suggests more factors are affected in the absence of *pink1*. There exist previous genetic studies where loss of *pink1* is rescued independently from *parkin* phenotypes such as Klein P, et.al., EMBO J. 2014. The complex interactions of all these genetic factors remain difficult to untangle for a comprehensive model of the *pink1-parkin* signaling axis *in vivo*.

2) Fig. 3C: I find surprising that the amount of p-ubiquitin increases in sting; park double mutants. An easy control for the hypothesis put forward by the authors lane 163 would be to include a loading control with a mitochondrial protein.

We agree with the reviewer. From some of the original samples and from new independent protein samples, we assayed the relative amount of the inner mitochondrial membrane protein ATP5α (Complex V subunit ATPsyn-α or *blw*). In the *pink1 5 allele* and both *parkin* mutants, a slightly decreased amount of ATP5α was observed (about 70-80% of wildtype). When adjusted for mitochondria protein content, the amount of pUB remains elevated in the double *sting; park* mutants, but the calculated degree of increase is reduced. We added this new data to the revised Figure S3. This suggests that there is a link between the amount of mitochondria and total phosphorylated Ub in these animals as well as additional factors related to p-Ubiquitin amounts. We do not know exactly where the pSer-65 Ub is localized in the absence of *parkin*. The results from Usher, J.L., el.al. EMBO Rep. 2022 suggest that p-Ser-65Ub may not be localized to the mitochondria aggregates in park-/- IFM tissues and its regulation is not dependent on the core autophagy components. Therefore, we think it is beyond the scope of this manuscript to determine the role of pSer65 in the *parkin* mutant phenotypes, however, these data do point out the value in further study of ubiquitin phosphorylation in context of Sting mutants and mitochondrial outer membrane regulation in Drosophila.

3) It is unfortunate that double mutant fly lines for parkin and Relish could not be generated. The explanation proposed by the authors (lane 178), which I doubt after looking at the evidence presented in the two articles cited, could easily be tested by raising the flies in axenic conditions or on antibiotics. A genetic interaction between Relish and parkin, even if opposite to the one between sting and parkin, would be interesting. To confirm an involvement of STING signaling in the parkin phenotype, the authors should target another component of the pathway, such as IKKb.

 To address this the *relish*, *parkin* mutant lines as well as *parkin* mutants and wildtype flies were raised on antibiotic-containing food for three generations, with two antibiotic cocktails (#1: 640 μg/ml doxycycline 640 μg/ml ampicillin, 1 mg/ml kanamycin, or #2: 500 μg/mL ampicillin, 50 μg/mL tetracycline, and 200 μg/mL rifamycin). Both of these protocols are reviewed in Lee HY, Aging 2019. From flies raised from embryos on antibiotic-containing food, no *parkin* homozygous flies eclosed, regardless of the *rel* mutant allele. In Parker-Character J, et.al. Sci Rep. 2021, similar antibiotic treatment of *parkin* flies also resulted in a noticeable decline in survival to adult eclosion. We are uncertain if this is due to toxicity of the antibiotics or an unknown interaction due to the absence of gut microbiota. See attached graph:

To address this second issue, we generated mutants for *IMD*[1]/CyO ; *park*[25]/TM6b. From these stocks, there were no viable *IMD/IMD; park/park* double mutants, similar to what was observed for *parkin* and *rel*[E20]. Flies that are heterozygous for *IMD*[1] and homozygous for *parkin25* have no change in penetrance of the *parkin* thorax phenotypes. We also crossed the *relE20, park25* to a *park25* stock and only recovered a low number of *rel*, *park25*/*park25* flies. These flies had severe parkin mutant phenotypes, with no suppression due to heterozygosity of *relish*, however as of this time, the number of flies is insufficient to include in this manuscript.

From these results, we conclude that *IMD* and *Relish* are synthetically lethal with *park*, and that haploinsufficiency of these genes does not robustly suppress *parkin* phenotypes. We also observed that in Fedele, G., et.al. *Nat Aging* **2**, 317–331 (2022) the authors make conclusions about rescue of *pink1* phenotypes solely from animals containing one mutant allele of *rel* or *eya*. We hypothesize that a similar lethality between innate immune genes and *pink1* might exist, but this remains untested.

4) Besides induction of a transcriptional response, STING can activate autophagy. Could a STING-dependent, possibly non canonical, autophagy explain the phenotype observed by the authors?

To test a role for STING-dependent non-canonical autophagy, like what was reported in Fischer et.al., we had generated a transgene for the bacterial effector SopF, which inhibits the ATG16/VTPase interaction with Atg16L1, and in mammalian cells, blocks STING-induced non-canonical autophagy. Expression of SopF with ubiquitous Gal4 drivers was developmentally lethal in flies. Overexpression of SopF via GMR-Gal4 resulted in severely deformed rough eyes, a phenotype commonly seen with cell death genes or with mis-regulation of protein homeostasis. Is seems that SopF, which ADP-ribosylates the V-ATPase in mammalian cells, is too toxic to express constitutively in animals. Therefore, this key technical tool to distinguish ATG8 lipidation in bulk autophagy from its role in non-canonical autophagy remain unavailable to us to use in vivo in the double mutant fly. Use of a tool like the GFP-RFP-Atg8 fusion protein would be necessary to further dissect the *in-vivo* dynamics of autophagy in the *sting* mutants and the *sting; parkin* double mutants, however we believe the genetics and time necessary for those experiments remains outside the scope of this manuscript.

We instead probed for accumulation of the autophagy receptor p62/ref(2)p in the parkin mutant flies, a previously described consequence of loss of the mitophagy machinery. In the *sting*; *parkin* double mutant animals, we observed an increase in the relative amount of p62 compared to single mutant controls (we note that these are young flies, about 4-days post-eclosion, so the p62 accumulation should not be due to the natural aging response) and these results are added now to Figure S3F & G. This may be due to prevention of p62 degradation or to increased expression of p62, which has previously been linked to pro-survival outcomes and increased longevity in flies. One possibility that unifies some of our observations is that p62 accumulation promotes a feed-forward activation loop of the NRF2 transcription factor, and upregulation of cell-protective oxidative stress responsive genes (See Aparicio, et.al. *Cell Reports* **28**, 2019 and Gumeni, S. et.al., *Cell Death Dis* 12, 671, 2021). We have revised the manuscript to reflect this finding and thank the reviewer for raising this possibility.

5) An obvious question raised by the data presented is whether the effect of STING is signal-dependent or constitutive? In other words, do cGAS-like receptors also contribute to the phenotype of parkin mutant flies? Is there an increase of cytosolic DNA or RNA in parkin mutant flies?

We had obtained an allele lacking both cGLR1 and cGLR2 on the 2^nd^ chromosome, and had outcrossed this allele into the *w*[1118]; ; *park*[1] background. Homozygous loss of both cGLR1 and cGLR2 did not suppress the bent wing posture phenotypes but did somewhat suppress the penetrance of the thorax indentation. We have added these data to Figure S4F of the revised manuscript, and this is reflected in the text (lines 203-207 and lines 286-290). Loss of these two cGas-like receptors does not rescue the phenotypes to the level that is observed in STING mutants. However, there remains additional cGas-like receptors in *Drosophila* species, according to recent preprints (Li., et.al. 2023 and Cai et.al. 2023). We have not completely ruled out the involvement of these additional receptors and the multitude of cyclic-di-nucleotide secondary messengers, but our data points toward a function of STING in the *parkin* mutant fly phenotype that may be independent of its activation canonical to the anti-viral response. This is similar to what was reported in *sting* mutants in both Akhmetova, K .et.al. 2021 and Rong, Y et.al. 2022. These studies’ data propose basal roles for STING in regulating homeostasis in pathways including autophagy or lipid metabolism. This is supported by our RNAseq results indicating loss of STING influences the cellular transcription response to oxidative stress, and that we do not see a robust induction of all the Sting-Regulated anti-viral factors in *parkin* flies. We currently lack tools to accurately quantify the release of mitochondrial DNA into the cytosol (or extracellular space) of the *parkin* mutant flies. Pilot IF experiments with anti-DNA antibodies in adult thorax muscles did not yield robust staining for non-nuclear DNA. Instead, we used multiplexed qPCR assays using Taqman-based probes against the mtDNA gene *CO:I* and observed a decrease in the relative copy number of mtDNA compared to nuclear DNA (*rpl32* probes). See our response to reviewer 1 for more details on these assays.

6) Minor comment: the order of the supplementary figures does not follow their appearance in the text (e.g., lane 163).

We have corrected the naming of the supplementary figures to reflect the order in the revised manuscript.

References:

Klein P, Müller-Rischart AK, Motori E, Schönbauer C, Schnorrer F, Winklhofer KF, Klein R. Ret rescues mitochondrial morphology and muscle degeneration of Drosophila Pink1 mutants. EMBO J. 2014 Feb 18;33(4):341-55. doi: 10.1002/embj.201284290. Epub 2014 Jan 28.

Usher, J.L., Sanchez‐Martinez, A., Terriente‐Felix, A., Chen, P., Lee, J.J., Chen, C., and Whitworth, A.J. (2022). Parkin drives pS65‐Ub turnover independently of canonical autophagy in *Drosophila*. EMBO Rep. 2022. 10.15252/embr.202153552.

Parker-Character J, Hager DR, Call TB, Pickup ZS, Turnbull SA, Marshman EM, Korch SB, Chaston JM, Call GB. An altered microbiome in a Parkinson's disease model Drosophila melanogaster has a negative effect on development. Sci Rep. 2021 Dec 8;11(1):23635. doi: 10.1038/s41598-021-02624-1

Fedele, G., Loh, S.H.Y., Celardo, I. *et al.* Suppression of intestinal dysfunction in a *Drosophila* model of Parkinson’s disease is neuroprotective. *Nat Aging* **2**, 317–331 (2022).

Lee HY, Lee SH, Lee JH, Lee WJ, Min KJ. The role of commensal microbes in the lifespan of *Drosophila melanogaster*. *Aging (Albany NY)*. 2019;11(13):4611-4640. doi:10.18632/aging.102073

Aparicio, R., Rana, A. & Walker, D. W. Upregulation of the Autophagy Adaptor p62/SQSTM1 Prolongs Health and Lifespan in Middle-Aged Drosophila. *Cell Reports* **28**, 1029-1040.e5 (2019).

Gumeni, S., Papanagnou, ED., Manola, M.S. et al. Nrf2 activation induces mitophagy and reverses Parkin/Pink1 knock down-mediated neuronal and muscle degeneration phenotypes. Cell Death Dis 12, 671 (2021)

Li Y, Slavik KM, Morehouse BR, de Oliveira Mann CC, Mears K, Liu J, Kashin D, Schwede F, Kranzusch PJ. cGLRs are a diverse family of pattern recognition receptors in animal innate immunity. *bioRxiv*. 2023 Feb 22:2023.02.22.529553. doi: 10.1101/2023.02.22.529553.

Hua Cai, Lihua Li, Kailey Slavik, et al. A novel virus-induced cyclic dinucleotide, 2′3′-c-di-GMP, mediates STING-dependent antiviral immunity in *Drosophila*. *bioRxiv*. 2023 May 8:2023.05.08.539652. doi:[10.1101/2023.05.08.539652](https://doi.org/10.1101/2023.05.08.539652)

Akhmetova, K., Balasov, M., and Chesnokov, I. (2021). Drosophila STING protein has a role in lipid metabolism. eLife *10*, e67358. 10.7554/elife.67358.

Rong, Y., Zhang, S., Nandi, N., Wu, Z., Li, L., Liu, Y., Wei, Y., Zhao, Y., Yuan, W., Zhou, C., et al. (2022). STING controls energy stress-induced autophagy and energy metabolism via STX17. J. Cell Biol. *221*, e202202060. 10.1083/jcb.202202060.
